# Four new species of *Symmerista* Hübner, 1816 (Notodontidae, Nystaleinae) from Costa Rica

**DOI:** 10.3897/zookeys.421.6342

**Published:** 2014-06-27

**Authors:** Isidro A. Chacón, Daniel H. Janzen, Winnie Hallwachs

**Affiliations:** 1 Instituto Nacional de Biodiversidad (INBio), Apdo. 22-3100, Santo Domingo de, Heredia, Costa Rica; 2 Department of Biology, University of Pennsylvania, Philadelphia, PA 19104, USA

**Keywords:** *Symmerista*, *Elymiotis*, *Elasmia*, Nystaleinae, moths, DNA barcodes, Neotropical, cloud forest, INBio, MINAE, Costa Rica

## Abstract

The genus *Symmerista* Hübner (Notodontidae, Nystaleinae) is reviewed for Costa Rica, based on 49 wild-caught specimens. Four species are newly described: *Symmerista luisdiegogomezi* Chacón, *Symmerista inbioi* Chacón, *Symmerista minaei* Chacón and *Symmerista aura* Chacón. All are from the cloud forests of the Talamanca moutain range, southern Costa Rica. Photographs of the adults, male and female genitalia, and barcodes are also provided. The species *Symmerista tlotzin* Schaus (1892) is removed from *Symmerista* and assigned to the genus *Elymiotis* Walker as a new combination.

## Introduction

The family Notodontidae includes about 3500 described species worldwide (Weller 1992). Approximately 896 species in 127 genera have been recorded in Costa Rica (Chacón et al. 2013). The subfamily Nystaleinae contains approximately 300 species and is restricted almost entirely to the Neotropics (Miller 1991), with a few species extending as far north as Canada (Forbes 1948). In Costa Rica, 191 Nystaleinae species are known to occur.

Hübner (1821) established the genus *Symmerista*, which until now has contained 12 described species (Franclemont 1946; Thiaucourt 2007; Thiaucourt and Monzón 2013). Species of *Symmerista* occur from Panama as far north as southeastern Canada. We have discovered four species of this genus in Costa Rica, found in the Talamanca Cordillera (Talamanca mountain range), from the cloud forests at altitudes between 1000 and 2600 meters.

Our studies of *Symmerista tlotzin* (Schaus, 1892) male genitalia morphology and barcodes (Janzen et al. 2009) suggest that it was improperly placed in that genus; here we assign *Symmerista tlotzin* to the genus *Elymiotis* Walker, 1857, as a new combination.

## Material and methods

Forty-nine specimens of *Symmerista* from the collection of the Instituto Nacional de Biodiversidad (INBio), Santo Domingo de Heredia, Costa Rica were examined, sexed and identified. Of those, 12 were DNA barcoded and dissected. All holotypes and paratypes are deposited in the collections at INBio. Fifty-six specimens of reared and wild-caught *Elymiotis tlotzin*, previously known as *Symmerista tlotzin*, were also examined.

### Repository abbreviations

INBio Instituto Nacional de Biodiversidad, Santo Domingo de Heredia, Costa Rica

USNM National Museum of Natural History, Smithsonian Institution, Washington DC, USA

### Key to morphological terminology

AD Adterminal line

CB Corpus bursae

DB Ductus bursae

FW Forewing

HW Hindwing

M Medial line

PM Postmedial line

ST8 Sternum 8

T8 Tergum 8

WL Wing length

## Systematics

### Symmerista

Taxon classificationAnimaliaLepidopteraNotodontidae

Hübner

Symmerista Hübner, 1821. Verz. bekannt. Schmett. : 248.

#### Type-species.

*Noctua albicosta* Hübner, 1809, Samml. eur. Schmett. 4: pl. 93, fig. 440. By subsequent designation by, Kirby, 1892, Syn. Cat. Lepid. Het. Het. 1: 572.

#### Type specimens.

Type(s), [North America]: Mistakenly included by Hübner as a European species; *Noctua albicosta* occurs in Canada and the US.

#### Notes.

Nye (1975) stated: *Symmerista* was originally proposed in the Noctuidae.

Watson et al. (1980) stated: *Noctua albicosta* is a form of *Phalaena albifrons* Smith, 1797, in Smith & Abbot, Nat. Hist. rarer lepid. Insects Georgia 2: 159, pl. 80.

#### Diagnosis.

***Adults*** – Medium-sized notodontid moth, FW = 16–22 mm, females larger than males; male antenna pectinate nearly to apex, terminal 10–12 annulations simple or antenna bipectinate, six terminal flagellomeres without rami; antennae of female simple; labial palpus porrect; haustellum reduced to two small lobes, completely hidden by labial palpi; ocelli absent; eye smooth, round; thorax generally dark brown with beige tegula; all scales of thorax long and forked; scales of patagium and prothorax beige and cream colored. FW with the costa straight, apex marked, the outer margin evenly rounded; accessory cell present, narrow; R_2-5_, stalked, R_5_ arising beyond R_2_, M_1_ from apex of accessory cell or nearly so. ***Male genitalia*** – ST8 with a wide, deep emargination; valva membranous, finely pubescent, costulae absent; tegumen narrowed dorsally; uncus concave, the socii subquadrate and lobed or long, wide, pubescent at bases, narrow and flattened at apices; vinculum slightly sclerotized; manica membranous, fused to juxta; ventral process of phallus long and forked, its tip well beyond the tip of the medial, dorsal process; vesica bulbous with a scobinate patch.

***Female genitalia*** – Papillae anales membranous, covered with short, scattered setae; posterior apophyses long and slender; DB sclerotized for approximately two-thirds of its length; CB rounded; anterior vaginal plate asymmetrical, slightly swollen and inflated in middle.

The genus *Symmerista* is characterized by: haustellum reduced to two small lobes, completely hidden by labial palpi; valva membranous; costulae absent; uncus concave; manica membranous, fused to juxta and ductus bursae sclerotized for approximately two-thirds of its length. *Symmerista* differs of the genera *Elasmia* and *Elymiotis* by the following characteristics: *Elasmia* has a well-developed haustellum, sacculus pleated and saccular scent organ present, manica sclerotized, deciduous cornuti, costulae present, costal margin sclerotized, ST8 with long and curvy lateral sclerotized projections like forceps, midplate with tiny pleats and membranous; uncus elongate, setose, with two dorsal protuberances and two tiny spines at the apex. *Elymiotis* has the haustellum well developed, sacculus pleated, deciduous cornuti, costal margin sclerotized, St8 posterior margin irregularly sclerotized and convex with tiny lateral proyections, uncus thin and long with apex acute and setose. All of these characterisctics are absent in *Symmerista*.

### Symmerista luisdiegogomezi

Taxon classificationAnimaliaLepidopteraNotodontidae

Chacón sp. n.

http://zoobank.org/81A8E740-7368-45A7-9BBE-6113BBC43D3E

Figs 1 –9

#### Material examined.

16 specimens (14 males, 2 females)

#### Type material.

**Holotype** male: INB0003536238 (dissected, COI barcoded), Costa Rica, Prov. Cartago, P.N. Tapanti, Macizo de La Muerte, Est. La Esperanza 9.69129-83.87683, 2600 m, September 2002, R. Delgado (INBio).

**Paratypes:** 13 males, 2 females. 2 males: INB0003756237, INB0003756364 Costa Rica, Prov. Limon, Parque Internacional La Amistad, Valle del Silencio, Alrededor del Refugio y el Sendero Circular, 9.110281-82.961934, 2450 m, 22–27 September 2003, D. Rubi, R. Gonzalez, R. Delgado (INBio). 4 males: INB0003316532, INB0003316533, INB0003316534, INB0003316535 Costa Rica, Prov. Cartago, El Guarco, Macizo de la Muerte, Sector La Esperanza, 9.686771-83.87775, 2600 m, June 2001, R. Delgado (INBio). Male: INB0003352709 Costa Rica, Prov. Cartago, Reserva Forestal Rio Macho. El Guarco, Macizo de la Muerte, Sector La Esperanza, 9.686771-83.87775, 2600 m, August 2001, R. Delgado (INBio). 2 males: INB0003387641 (dissected), INB0003387642, Costa Rica, Prov. Cartago, Reserva Forestal Rio Macho, El Guarco, Macizo de la Muerte, 9.686771-83.87775, 2600 m, October 2001, R. Delgado (INBio). 2 males: INB0003536234 (COI barcoded), INB0003536235 (COI Barcoded), INB0003536237 (COI barcoded), Costa Rica, Prov. Cartago, Parque Nacional Tapanti, Macizo de La Muerte, Est. La Esperanza, 9.69129-83.876832, 2600 m, September 2002, R. Delgado (INBio). 2 males: INB0003545221 (dissected, COI barcoded), INB0003545222 (COI barcoded) Costa Rica, Prov. Cartago, Parque Nacional Tapanti, Macizo de La Muerte, Est. La Esperanza, 9.69129-83.876832, 2600 m, October 2002, R. Delgado (INBio). Female: INB0003339209 (dissected, COI barcoded) Costa Rica, Prov. Cartago, El Guarco, Macizo de la Muerte, Est. La Esperanza. 9.686771-83.87775, 2600 m. July 2001. R. Delgado (INBio). Female: INB0003756274 Costa Rica, Prov. Limon, Parque Internacional La Amistad, Valle del Silencio, Alrededor Refugio y Sendero Circular, 9.110281-82.961934, 2450 m, 22–27 September 2003, D. Rubi, R. Gonzalez, R. Delgado (INBio).

#### Etymology.

This species is named in honor of the late Professor Luis Diego Gómez Pignataro of San Jose, Costa Rica, for his outstanding contribution to our knowledge of Costa Rican biodiversity, his support of the Museo Nacional de Costa Rica, and for inspiring me to become a naturalist and taxonomist.

#### Diagnosis.

Dorsal FW ground color dark brown with costal margin black; an irregular, long thin cream-colored mark from the reniform spot to the apex; fringe dark brown with beige scales where veins touch termen; dorsal HW dark brown. **Male genitalia:** St8 posterior margin concave with a pair of short postero-lateral projections, projections robust and heavily sclerotized with blunt apices; valva membranous, finely pubescent, sacculus smooth, its margin uniform, costa straight with a distal protuberance near apex; valva with two triangular spine-like processes, one at base, the other at juxta near anellus; uncus slightly concave, somewhat helmet shaped, dorsal surface rough, pubescent, ventral surface smooth with sparse pubescence; socii long, wide, pubescent at bases, narrow and flattened at apices, shape as in the figure; length of the phallus 3.1 mm, proximal part of phallus tube wide at the base, narrow in the middle, distal part of phallus tube robust, sclerotized, with a tubular lateral projection and rounded apex; proximal part of the vesica with a ventral scobinate patch, distal part of the vesica bulbous. **Female genitalia:** posterior apophyses longer and more slender than anterior apophyses; CB rounded and membranous, lacking a signum; DB wide and sclerotized; posterior margin of postvaginal plate sclerotized and emarginate.

#### Description.

**Figures 1–4.**
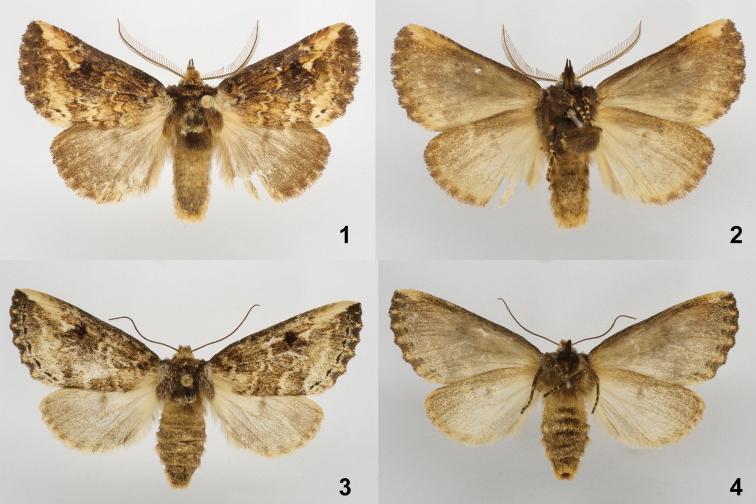
*Symmerista luisdiegogomezi* **1, 2** Holotype male dorsal and ventral INB0003536238 **3, 4** Paratype female dorsal and ventral INB0003339209.

**Figures 5–8.**
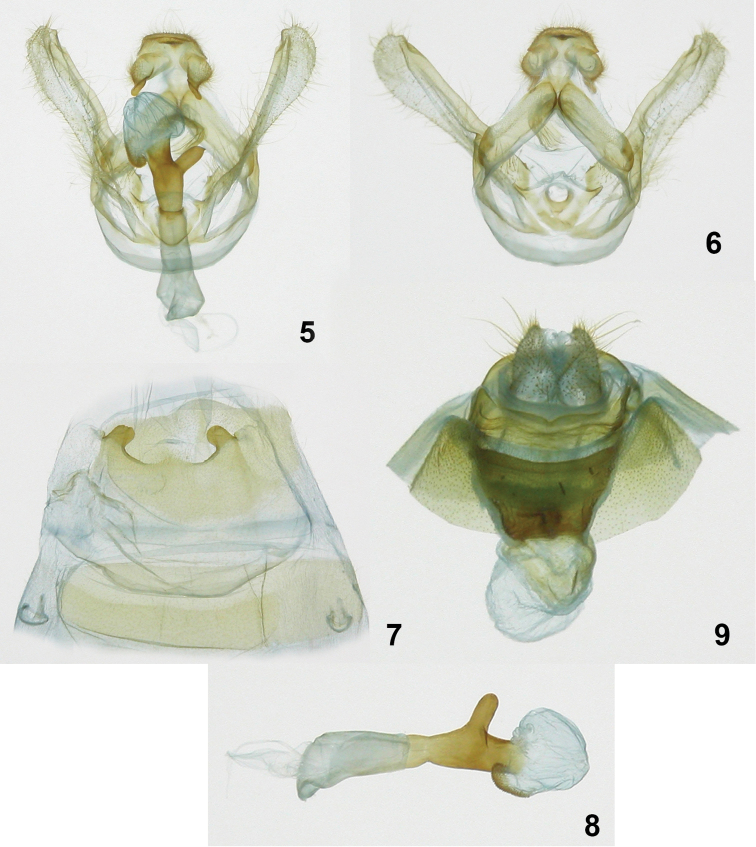
*Symmerista luisdiegogomezi* **5, 6** Holotype male genitalia INB0003536238 **7** Male St8 **8** Phallus **9** Paratype female genitalia INB0003339209.

**Male** (Figs 1, 2, 5–8). ***Head*** – Antenna bipectinate, dark brown, six terminal flagellomeres without rami, antennal shaft dark brown dorsally and beige ventrally; scape bearing a long tuft of beige scales; haustellum reduced to two small lobes; eye smooth, round, black; frons mostly dark brown and black with brown-yellow scales; labial palpus porrect, dark brown ventrally, light brown dorsally; vertex brown yellow; patagium light brown. ***Thorax and abdomen*** – Tegula dark brown at base, a mix of cream and dark brown scales distally; mesoscutellum dark brown; thoracic pleuron from creamy white to dark brown; dorsal area of metathorax with black hair-like scales; legs mostly dark brown with cream-colored scales between segments; abdominal dorsum dirty beige, venter beige. ***Wings*** – Dorsal FW ground color dark brown, with reniform spot black; basal band light brown; M sinuous, light brown; postmedial band light brown; an irregular, thin cream-colored bar from reniform spot to apex; AD black; fringe dark brown with beige scales where veins touch termen; a light beige area between subterminal line and postmedial band of M_3_ extending to tornus; dorsal HW dark brown; fringe dark brown with beige scales where veins touch termen (Figs 1, 2) (WL 17.20–19.21 mm). ***Genitalia*** (Figs 5–8) – T8 wider than long, with two windows, anterior margin slightly concave, posterior margin sclerotized; St8 wide at base, narrowing posteriorly, posterior margin concave with tiny sclerotized teeth on edge, with a pair of short lateral projections, these sclerotized with blunt apices (Fig. 7); valva membranous, finely pubescent, costulae absent, sacculus smooth, its margin uniform, costa straight with a distal protuberance near apex; valva with two triangular spine-like processes, one at base, other at juxta near anellus; tegumen narrowed dorsally; uncus slightly concave, somewhat helmet shaped, dorsal surface rough, pubescent, ventral surface smooth with sparse pubescence; socii long, wide, pubescent at bases, narrow and flattened at apices, shape as in the figure; juxta ovoid; vinculum lightly sclerotized (Fig. 5); length of phallus 3.1 mm, proximal part of phallus tube wide at base, narrow in middle, distal part of phallus tube robust, sclerotized, with a tubular lateral projection and rounded apex; proximal part of vesica with a ventral scobinate patch, distal part of the vesica bulbous (Fig. 8).

**Female** (Figs 3, 4, 9) Similar to the male: ***Head*** – Antenna filiform, antennal shaft dark brown with cream scales, scape bearing a long tuft of cream scales; frons and vertex dark brown; haustellum vestigial; labial palpus dark brown. ***Thorax and abdomen*** – Mostly dark brown, tegula beige; dorsal area of metathorax with black hair-like scales; abdominal dorsum light brown, venter beige. ***Wings*** – Dorsal FW ground color dark brown; antemedial band beige, lined on both sides by sinuous dark brown lines; irregular white thin bar from apex to reniform spot; reniform spot black; postmedial band beige, subterminal line black; a light beige area between subterminal line and postmedial band of M_3_ extending to tornus; fringe dark brown with beige scales located where veins touch termen. Dorsal HW dirty beige; postmedial line light brown; discal spot black; fringe dark brown with beige scales located where veins touch termen (Figs 3, 4) (WL 20.22–21.51 mm). ***Genitalia*** (Fig. 9) – Papillae anales slightly ovoid, setose; posterior apophyses longer and more slender than anterior apophyses; DB wide and sclerotized; CB rounded and membranous, lacking a signum; posterior margin of postvaginal plate sclerotized and emarginate.

#### Distribution and habitat.

*Symmerista luisdiegogomezi* has been collected only between 2450 and 2600 m in the highland cloud forests dominated by *Quercus* trees in the foothills west of the Cordillera de Talamanca (Talamanca Mountain Range), southern Costa Rica (Fig. 49).

#### Remarks.

DNA barcode of male holotype INB0003536238.

MHMXP012-08 | INB0003536238 | *Symmerista luisdiegogomezi* | COI-5P

ACATTATATTTCATTTTTGGAATTTGAGCAGGTATAGTTGGAACTTCATTAAGCCTATTAATTCGAGCTGAATTAGGAAATCCAGGATCCCTTATTGGGGATGATCAAATTTATAATACAATTGTTACAGCCCATGCCTTTATTATAATTTTTTTTATAGTAATACCTATTATAATTGGGGGATTTGGTAATTGATTAATTCCCCTTATATTAGGGGCCCCAGACATAGCATTCCCACGTATAAATAATATAAGTTTTTGACTTTTACCCCCCTCCTTAACCCTTTTAATTTCAAGAAGAATTGTAGAAAATGGGGCAGGAACTGGATGAACAGTGTACCCCCCACTATCATCCAATATTGCTCATAGTGGAAGTTCTGTAGATTTAGCTATTTTTTCCCTTCATTTAGCTGGAATTTCATCAATTTTAGGGGCCATTAATTTTATTACAACAATTATTAATATACGTCTCAATAATATATCCTTTGATCAAATACCTTTATTTGTTTGGGCTGTTGGGATTACAGCATTTTTACTTTTACTTTCTTTACCTGTTTTAGCTGGAGCTATTACAATACTACTAACTGATCGAAATTTAAACACATCTTTTTTTGACCCTGCAGGAGGGGGAGATCCAATTTTATATCAACATTTA

### Symmerista inbioi

Taxon classificationAnimaliaLepidopteraNotodontidae

Chacón sp. n.

http://zoobank.org/72BC7743-A984-4260-8CD8-CF7E47FDB9AA

Figs 10 –16

#### Material examined.

21 specimens (21 males).

#### Type material.

**Holotype** male: INB0003487050 (dissected, COI barcoded), Costa Rica, Prov. Cartago, El Guarco, Reserva Forestal Rio Macho, Macizo de la Muerte, Sector La Esperanza 9.686771-83.87775, 2600 m, May 2002, R. Delgado (INBio).

**Paratypes:** Male: INB0003153991 Costa Rica, Prov. Cartago, El Guarco, San Isidro, Est. La Esperanza 9.687685-83.884582, 2450 m, March 2001, R. Delgado (INBio). 2 males: INB0003316531, INB0003316537 Costa Rica, Prov. Cartago, El Guarco, Macizo de la Muerte, Sector La Esperanza, 9.686771-83.87775, 2600 m, June 2001, R. Delgado (INBio). Male: INB0003320716 Costa Rica, Prov. Cartago, Parque Nacional Tapanti, El Guarco, San Isidro, Est. La Esperanza, 9.683922-83.876688, 2600 m, May 2001, R. Delgado (INBio). Male: INB0003334468 Costa Rica, Prov. Cartago, Parque Nacional Tapanti, Macizo de la Muerte, Est. La Esperanza, 9.686771-83.87775, 2600-2700 m, April 2001, R. Delgado (INBio). 3 males: INB0003339195, INB0003339197, INB0003339198 Costa Rica, Prov. Cartago, El Guarco, Parque Nacional Tapanti, Macizo de la Muerte, Est. La Esperanza 9.686771-83.87775, 2600 m, July 2001, R. Delgado (INBio). Male: INB0003387640 Costa Rica, Prov. Cartago, Reserva Forestal Rio Macho, El Guarco, Macizo de la Muerte, 9.686771-83.87775, 2600 m, October 2001, R. Delgado (INBio). Male: INB0003478225 (dissected, COI barcoded), Costa Rica, Prov. Cartago, Parque Nacional Tapanti, Macizo de La Muerte, Est. La Esperanza, 9.69397-83.854504, 2700 m, 13–14 May 2002, J. Montero (INBio). 2 males: INB0003536236 (dissected, COI barcoded), INB0003536239 (COI Barcoded) Costa Rica, Prov. Cartago, Parque Nacional Tapanti, Macizo de La Muerte, Est. La Esperanza, 9.69129-83.876832, 2600 m, September 2002, R. Delgado (INBio). Male: INB0003545219 (dissected, COI barcoded) Costa Rica, Prov. Cartago, Parque Nacional Tapanti, Macizo de La Muerte, Est. La Esperanza, 9.69129-83.876832, 2600 m, October 2002, R. Delgado (INBio). 3 males: INB0003756229, INB0003756321, INB0003756418 Costa Rica, Prov. Limon, Parque Internacional La Amistad, Valle del Silencio, Alrededor del Refugio y Sendero Circular, 9.110281-82.961934, 2450 m, 22–27 September 2003, D. Rubi, R. Gonzalez, R. Delgado(INBio). Male: INBIOCRI001359567 Costa Rica, Prov. Cartago, Quebrada Segunda, Parque Nacional Tapanti, 9.762583-83.788328, 1250 m, February 1993, G. Mora. Male: INBIOCRI002210601 Costa Rica, Prov. Cartago, La Represa, Tapanti, 9.695643-83.768399, 1800 m, July 1995, R. Delgado (INBio). Male: INBIOCRI002253344 Costa Rica, Prov. Cartago, Rio Grande de Orosi, desde Puente Rio Dos Amigos hasta la Represa, 9.695643-83.768399, 1400-1800 m, March 1995, R. Delgado (INBio). Male: INBIOCRI002423774 Costa Rica, Prov. Cartago, Rio Grande de Orosi, desde Puente Rio Dos Amigos hasta la Represa, 9.695643-83.768399, 1800 m, February 1995, R. Delgado (INBio). Male, INBIOCRI002427627 Costa Rica, Prov. Cartago, Rio Grande de Orosi, desde Puente Rio Dos Amigos hasta la Represa, 9.695643-83.768399, 1400-1800 m, 22 August–15 September 1995, R. Delgado (INBio).

#### Etymology.

This species is dedicated to the Instituto Nacional de Biodiversidad (INBio) in recognition of its 25 years of support for developing an understanding of the biodiversity of Costa Rica and exporting that understanding to the nation and the world.

#### Diagnosis.

*Symmerista inbioi* differs from *Symmerista luisdiegogomezi* on: dorsal FW ground color light brown; from the reniform spot to the apex there is an uniform long, thin, white cream-colored band; fringe reddish brown; dorsal HW dirty beige. **Male genitalia:** St8 wide at base, anterior margin convex, posterior margin slightly irregular with a pair of very short projections, these sclerotized with blunt apices; valva membranous, saccular margin slightly jagged, costal margin smooth, with a distal protuberance near the apex; elongate finger-shaped process on the saccular margin at the internal base of the valve; uncus plate slightly concave, with papillae and setae on the dorsal and ventral edge; socii elongated, broad at base, narrow and flattened at the apex, with papillae and setae in the dorsal surface; length of the phallus 3.9 mm, proximal part of phallus curved at the base, slightly narrow in the middle, distal part of phallus tube sclerotized, narrow at the base, robust, irregular and wide at the end, with a tubular lateral projection with the distal nipple; proximal part of the vesica with a dorsal scobinate patch, distal part of the vesica bulbous. The genital armature of *Symmerista inbioi* is more robust and larger than *Symmerista luisdiegogomezi*.

#### Description.

**Figures 10, 11.**
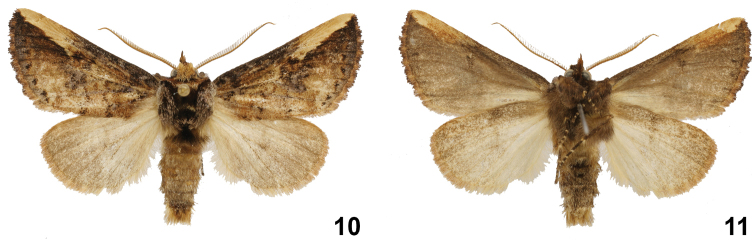
*Symmerista inbioi* Holotype male dorsal and ventral INB0003487050.

**Figures 12–16.**
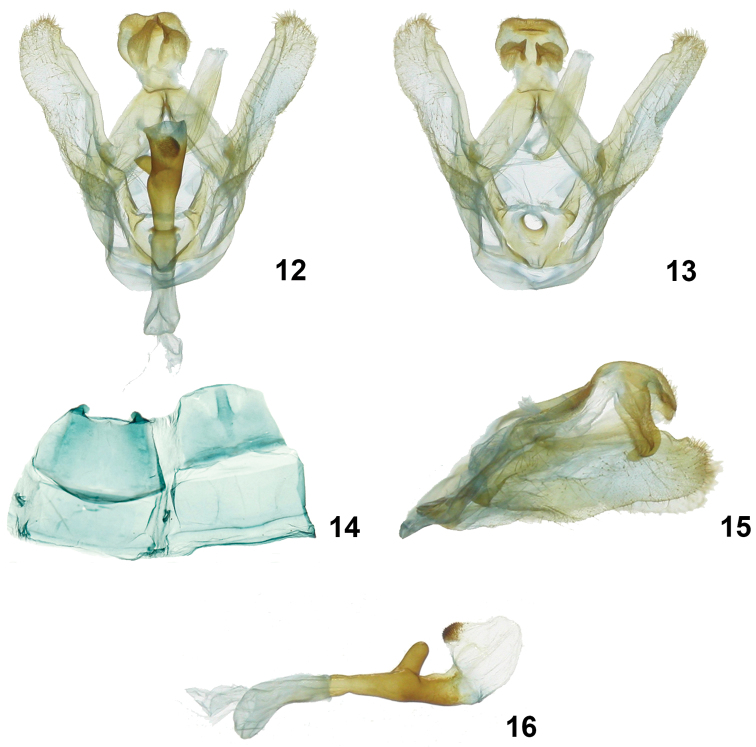
*Symmerista inbioi* **12, 13** Holotype male genitalia INB0003487050 **14** Male St8 **15** Uncus **16** Phallus.

**Male** (Figs 10, 11, 12–16). ***Head*** – Antenna bipectinate nine terminal flagellomeres without rami, antenal shaft cream dorsally and light brown ventrally, scape bearing a long tuft of yellow-brown scales; haustellum vestigial; eye smooth, round, black; frons dark brown; labial palpus porrect, brown; patagium light brown near midline, dark brown laterally. ***Thorax and abdomen*** – Tegula dark brown at base, a mix of cream and dark brown scales distally; mesoscutellum black; thoracic pleuron dark brown; legs dark brown with cream-colored scales between segments; abdominal dorsum light brown, venter dark brown.

***Wings*** – Dorsal FW ground color light brown; with reniform spot black; from reniform spot to apex with a uniform, long, thin, creamy-white band; costal margin black until R_1_; AD black; purple scales between terminal line and the AD; fringe reddish brown; dorsal HW dirty beige, fringe light brown (Figs 10, 11) (WL 16.42–19.44 mm). ***Male genitalia*** (Figs 12–16) – T8 rectangular, wider than long, posterior margin slightly sclerotized and serrated; St8 wide at base, anterior margin convex, posterior margin slightly irregular with a pair of very short projections, these sclerotized with blunt apices (Fig. 14); valva membranous, costulate absent, saccular margin slightly jagged, costal margin smooth, with a distal protuberance near apex; elongate finger-shaped process on saccular margin at internal base of valve; tegumen narrow at point of intersection of both arms, margins heavily sclerotized (Figs 12, 13); uncus plate concave, with papillae and setae on dorsal and ventral edge; socii elongated, broad at base, narrow and flattened at apex, with papillae and setae in dorsal surface (Figs 12, 13, 15); juxta heart shaped (Fig. 13); vinculum membranous, slightly sclerotized (Fig. 12); length of phallus 3.9 mm, proximal part of phallus curved at base, slightly narrow in middle, distal part of phallus tube sclerotized, narrow at base, robust, irregular and wide at end, with a tubular lateral projection with distal nipple; proximal part of vesica with a dorsal scobinate patch, distal part of vesica bulbous (Fig. 16).

**Female:** unknown

#### Distribution and habitat.

*Symmerista inbioi* has been collected only at elevations betwen 1250 and 2700 m in highland cloud forests of the Cordillera de Talamanca (Talamanca Mountain Range) (Fig. 49).

#### Remarks.

DNA barcode of holotype male INB0003487050

MHMXP003-08 | INB0003487050 | *Symmerista inbioi* | COI-5P:

AACATTATATTTTATTTTTGGGATTTGAGCAGGTATAGTAGGAACTTCTTTAAGTCTATTAATTCGAGCTGAATTAGGAAACCCCGGATCACTTATTGGGGATGATCAAATTTATAATACAATTGTTACAGCCCATGCCTTTATTATAATTTTTTTTATGGTAATACCTATTATAATTGGGGGATTTGGTAATTGATTAGTCCCTCTTATACTAGGAGCCCCAGATATAGCATTCCCCCGCATAAATAATATAAGTTTTTGACTTTTGCCCCCTTCTTTAACCCTTTTAATTTCAAGAAGAATCGTAGAAAATGGAGCAGGAACTGGATGGACAGTGTACCCCCCACTATCCGCCAACATTGCCCATAGTGGAAGTTCTGTAGATTTAGCTATTTTTTCCCTTCATTTAGCTGGAATTTCCTCAATTTTAGGAGCTATTAATTTTATTACAACAATTATTAATATACGCCTCAATAATATATCTTTTGATCAAATACCTTTATTTGTTTGAGCTGTTGGAATTACAGCATTTTTACTTTTACTTTCTTTACCTGTTTTAGCGGGAGCTATTACAATACTACTAACTGACCGTAATTTAAATACATCCTTTTTTGACCCTGCTGGGGGAGGAGATCCAATTTTATACCAACATTTATTT

### Symmerista minaei

Taxon classificationAnimaliaLepidopteraNotodontidae

Chacón sp. n.

http://zoobank.org/71A2F18E-A8EC-4BF6-9991-ACEC39D20799

Figs 17 –25

#### Material examined.

4 specimens (1 male, 3 females)

#### Type material.

**Holotype** female: INB0003339208 (dissected, COI barcoded), Costa Rica, Prov. Cartago, El Guarco, Macizo de la Muerte, Estacion La Esperanza, 9.68677-83.87775, 2600 m, July 2001, R. Delgado (INBio). **Paratypes:** Female: INB0003155283 (COI barcoded), Costa Rica, Prov. Limon, Bratsi, Valle del Silencio, 9.107197-82.961749, 2472 m, 11–12 October 2000, R. Delgado (INBio). Female: INB0003352700 (COI barcoded), Costa Rica, Prov. Cartago, Reserva Forestal Rio Macho, El Guarco, Macizo de la Muerte, Sector La Esperanza, 9.686771-83.87775, 2600 m, August 2001, R. Delgado (INBio).

Other material examined: 1 Male, INB00033387642 (dissected) Costa Rica, Prov. Cartago, Reserva Forestal Rio Macho, El Guarco, Macizo de la Muerte, 9.68677-83.87775, 2600 m, October 2001. R. Delgado (INBio).

#### Etymology.

This species is dedicated to the Ministerio del Ambiente y Energía (MINAE) of the government of Costa Rica in recognition of its 28 years of continuous and widespread support for the survival and conservation of the wild biodiversity of Costa Rica.

#### Diagnosis.

*Symmerista minaei* differs from *Symmerista luisdiegogomezi* on: dorsal FW ground color beige and light brown, square mark creamy near the reniform spot; beige mark in the apex; fringe beige yellow; dorsal HW beige. **Male genitalia:** T8 anterior margin slightly concave, posterior margin finely serrated with a window in the center; St8 lateral margins wide at the base, narrow to posterior margin, anterior margin concave, slightly sclerotized with a short projection in the center, posterior margin with robust projections, highly sclerotized on each side, with blunt apices, a little dome in the middle of the posterior margin; length of the phallus 3.3 mm, proximal part of phallus tube wide at base, distal part of phallus tube robust, sclerotized, with a tubular lateral projection rounded apex with the distal nipple; proximal part of the vesica with a ventral scobinate patch, distal part of the vesica bulbous. **Female genitalia:** Anterior and posterior apophyses the same size, long an slender; DB sclerotized; CB rounded, membranous and pleated; posterior margin of postvaginal plate sclerotized, slightly irregular, inverted V-shape.

#### Description.

**Figures 17–20.**
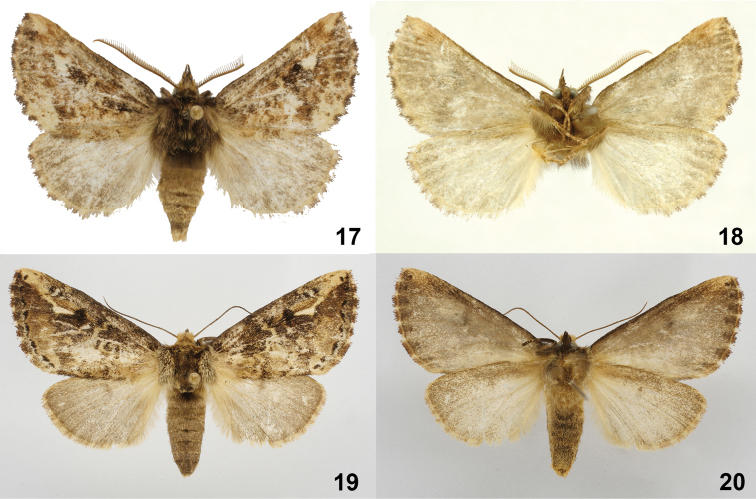
*Symmerista minaei* **17, 18** Paratype male dorsal and ventral INB0003387642 **19**, **20** Holotype female dorsal and ventral INB0003339208.

**Figures 21–25.**
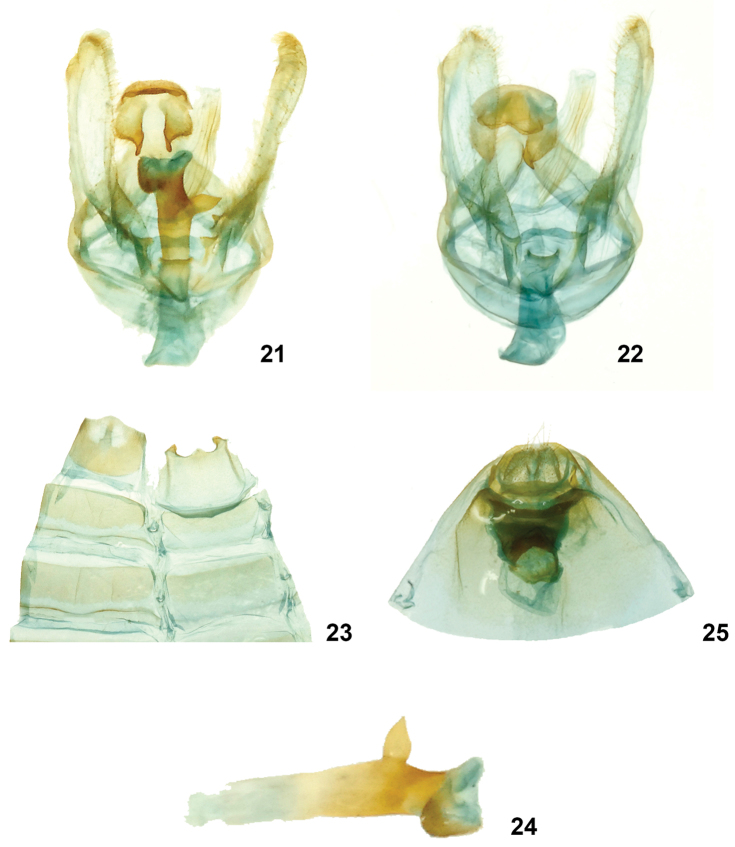
*Symmerista minaei* INB0003387642 **21, 22** Paratype male genitalia **23** Male St8 **24** Phallus **25** Holotype female genitalia INB0003339208,

**Male** (Figs 17, 18, 21–24). ***Head*** – Antenna bipectinate, dark brown, six terminal flagellomeres with very short rami, antennal shaft brown dorsally and light brown ventrally; scape bearing a long tuft of beige scales, sensilla beige; eye smooth, round, black; frons mostly dark brown and black with beige scales; labial palpus porrect, dark brown ventrally, light brown dorsally; vertex beige with black scales; patagium dark brown and light brown.

***Thorax and abdomen*** – Tegula dark brown at base, a mix of dark brown and light brown scales distally; mesoscutellum beige and dark brown; thoracic pleuron from beige to dark brown; dorsal area of metathorax with black and dark brown hair-like scales; legs mostly dark brown with beige scales between segments; abdominal dorsum dirty beige, venter beige. ***Wings*** – dorsal FW ground color beige and light brown, with reniform spot black; basal band light brown; postmedial band light brown; a creamy-white square distal to reniform spot; AD black; fringe dark brown with beige scales where veins touch termen; beige mark in apex; dorsal HW beige; fringe beige-yellow (Figs 17, 18) (WL 17.20–19.21 mm). ***Male genitalia*** (Figs 21–24) – T8 wider than long, anterior margin slightly concave, posterior margin finely serrated with a window in center; St8 lateral margins wide at base, narrow to posterior margins, anterior margin convex, slightly sclerotized with a short projection in center, posterior margin concave with a pair of short projections, these sclerotized with blunt apices, a small setose dome in middle of posterior margin (Fig. 23); valva membranous, mildly pubescent, margin of sacculus slightly serrated, costa with straight margin with a distal protuberance close to apex; valva with a triangular spine-like process; tegumen narrowed dorsally; uncus plate slightly concave, somewhat helmet shaped, dorsal surface rough, pubescent, ventral surface smooth with sparse pubescence, socii long, wide, pubescent at bases, narrow and flattened at apex, form s-shaped; vinculum slightly sclerotized (Figs 21, 22); length of phallus 3.3 mm, proximal part of phallus tube wide at base, distal part of phallus tube robust, sclerotized, with a tubular lateral projection rounded apex with distal nipple; proximal part of vesica with a ventral scobinate patch, distal part of vesica bulbous (Fig. 24). **Female** (Figs 19, 20, 25) ***Head*** – Antenna simple, shaft dark brown with cream-colored scales, scape with a tuft of cream-colored scales; eyes naked; frons dark brown, vertex dark brown with groups of beige scales at base and between antennal bases; haustellum vestigial, labial palpus dark brown.

***Thorax and abdomen*** – Generally dark brown, tegula beige, scales of thorax long and forked. Patagium and prothorax with beige and cream scales; mesothorax with scales cream, dark brown and beige; metathorax with a group of black hair-like scales along posterior margin; abdomen light brownish gray, abdominal apex with a thick group of beige scales.

***Wings*** – dorsally FW ground color dark brown; antemedial beige band lined at both sides by sinuous dirty dark brown lines; reniform spot black; an irregular thin white to beige line extends from the apex to the reniform spot; postmedial line beige, lined on each side with dark brown; adterminal line black; light beige area between adterminal line and postmedial band from M3 to tornus; fringe dark brown with beige scales where veins touch termen; dorsal HW dirty beige, fringe beige (Figs 19, 20) (WL 19.63–21.18 mm). ***Female genitalia*** (Fig. 25) – Papillae anales mambranous with short, scattered setae with longer, inwardly-curved setae arising from base. Anterior and posterior apophyses of same size, long and slender; DB sclerotized; CB rounded, membranous and pleated; posterior margin of postvaginal plate sclerotized, slightly irregular, inverted V-shape.

#### Distribution and habitat.

*Symmerista minaei* has only been collected at elevations between 2400 and 2600 m in highland cloud forests of the Cordillera de Talamanca (Talamanca Mountain Range) (Fig. 49).

#### Remarks.

DNA barcode paratype female INB0003155283.

MHMXP006-08 | INB0003155283 | *Symmerista minaei* | COI-5P:

AACATTATATTTCATTTTTGGAATTTGAGCAGGTATAGTTGGAACTTCATAAGCCTATTAATTCGAGCTGAATTAGGAAATCCCGGATCCCTTATTGGAGATGATCAAATTTATAACACAATTGTTACAGCCCATGCCTTTATTATAATTTTTTTTATAGTAATACCTATTATAATTGGGGGATTTGGTAATTGATTAGTCCCCCTTATGCTAGGAGCCCCAGATATAGCATTCCCACGTATAAATAATATAAGTTTTTGACTTTTACCCCCCTCCTTAACCCTTTTAATTTCAAGAAGAATCGTCGAAAATGGGGCAGGAACCGGATGGACAGTGTACCCCCCACTATCCTCCAATATTGCCCACAGTGGAAGTTCTGTAGATTTAGCTATTTTTTCCCTACATTTAGCTGGAATTTCATCAATTTTAGGGGCCATTAATTTTATCACAACAATTATTAATATACGTCTCAATAACATATCTTTTGATCAAATACCCTTATTTGTTTGAGCTGTTGGAATTACAGCATTTTTACTTTTACTTTCTTTACCTGTTCTAGGGAGCTATTACAATACTACTAACGGATCGTAATTTAAATACATCTTTTTTTGATCCTGCAGGAGGAGGAGATCCAATTTTATATCAACATTTATTT

### Symmerista aura

Taxon classificationAnimaliaLepidopteraNotodontidae

Chacón sp. n.

http://zoobank.org/31DCFCFA-B792-406E-A667-CAAA17B5111F

Figs 26 –35

#### Material examined.

4 specimens (2 males, 2 females)

#### Type material.

**Holotype** male: INB0003116415 (dissected, COI barcoded), Costa Rica, Prov. Cartago, Paraiso, P.N. Tapanti, Macizo de La Muerte, Estacion Quebrada Segunda, 9.762583-83.788328, 1300 m, November 2000, R. Delgado (INBio).

**Paratypes:** 1 males, 2 females. Male: INBIOCRI002442681 (COI barcoded), Costa Rica, Prov. Puntarenas, Buenos Aires, Potrero Grande, Estacion Altamira, 1 Km. S del Cerro Biolley, 9.032987-83.010887, 1450 m, 13–26 May 1996, R. Villalobos (INBio). Female: INBIOCRI002549508 (COI barcoded), Costa Rica, Prov. Puntarenas, Coto Brus, Sabalito, Send. El ripario a 3 Km NE. de Progreso, 8.917676-82.78469, 1300 m, 6–9 April 1997, A. Picado (INBio). Female: INBIOCRI002754030 Costa Rica, Prov. Cartago, Turrialba, Tayutic, Moravia de Chirripo Shipiri, 9.837781-83.453639, 1000 m, 10 May 1983, D. H. Janzen & W. Hallwachs (INBio).

#### Etymology.

This species is dedicated to Isidro Chacón’s daughter, Aura Chacón, for 25 years of understanding an absent father obsessed with his work.

#### Diagnosis.

Dorsal FW ground color light gray; a white cream mark from the discal cell to the apex. **Male genitalia:** St8 anterior margin with a sclerotized triangular projection in the middle, posterior margin sclerotized, irregular, with a rectangular projection at the center bearing two highly sclerotic and sharply serrated structures; proximal part of phallus tube wide and short at the base, with a blade-like lateral projection, sharp at the distal apex; a small rounded projection and curve off the previous, distal part of phallus tube robust, sclerotized, with a small bulge on the ventral side; proximal part of the vesica with a ventral scobinate patch, distal part of the vesica bulbous. **Female genitalia:** papillae anales ovoid-triangular, setose; posterior apophyses longer and more slender than anterior apophyses; DB sclerotized; CB elongated, membranous and pleated, lacking a signum; margin distal of antevaginalis plate very sclerotized and lightly depressed at the center, concave to the sides, proximal margin convex.

The female of *Symmerista aura* differs from female of *Symmerista meridionalis* Thiaucourt, 2007 in the shape of the antivaginalis plate; the CB elongated, membranous and pleated, lacking a signum; margin distal of antevaginalis plate very sclerotized and lightly depressed at the center, concave to the sides, proximal margin convex. This is the description of the female genitalia of *Symmerista meridionalis* published by Thiaucourt in 2007 which mentiones the differences, “Female terminalia: distal edge of lamella postvaginalis slightly incurved; lamella antevaginalis rectangular, almost square; its distal margin strongly sclerotized; mouth of the ostium bursae oval, densely sclerotized under the margin; ductus bursae beyond the constriction under the ostium, forming a short funnel; bursa membranous inserted on the dorsal surface of the extremity of the duct; signum distinct.” The male of *Symmerista meridionalis* is unknown.

#### Description.

**Figures 26–29.**
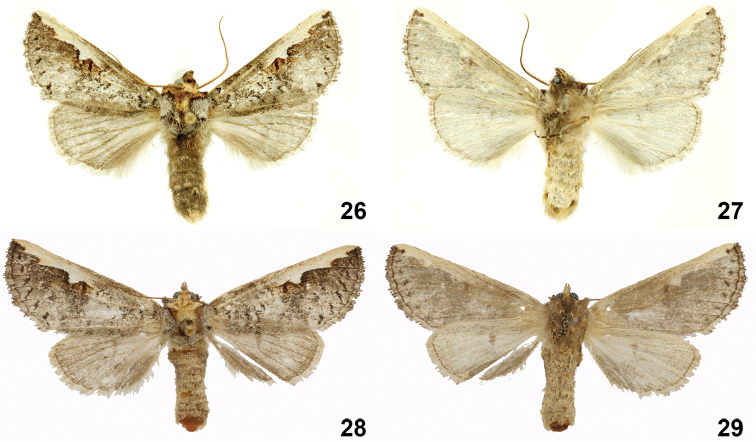
*Symmerista aura* **26, 27** Paratype male dorsal and ventral INBIOCRI002442681 **28, 29** Paratype female dorsal and ventral INBIOCRI002549508.

**Figures 30–35.**
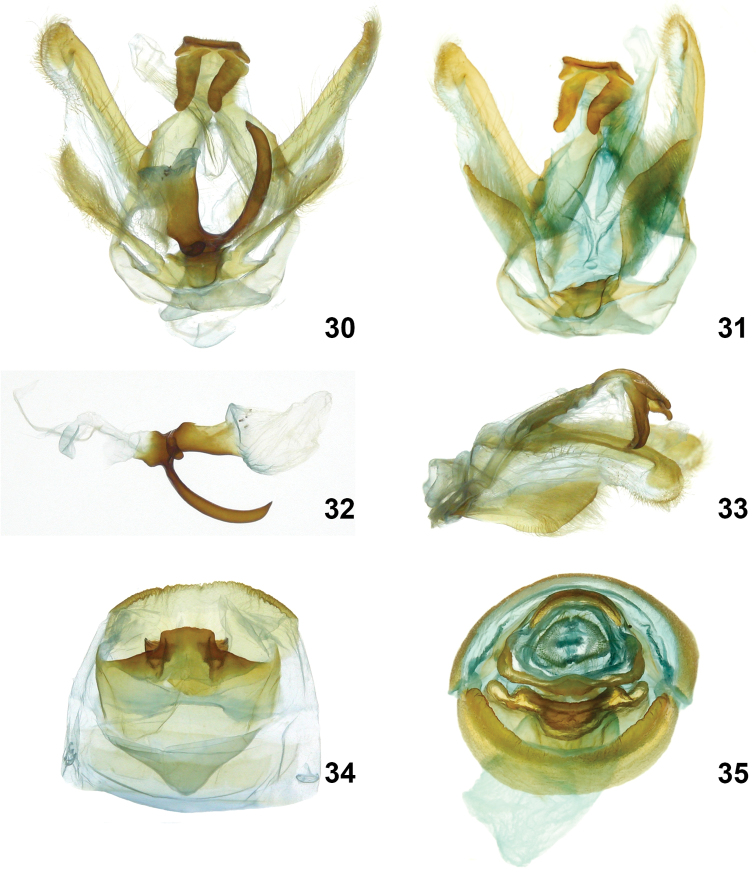
*Symmerista aura* INB0003116415 **30, 31** Holotype male genitalia **32** Phallus **33** Uncus **34** Male St8 **35** Paratype female genitalia INBIOCRI002549508.

**Male** (Figs 26, 27, 30–34) ***Head*** – antenna bipectinate, ventral shaft dark brown, dorsal light brown, scape bearing a tuff of beige scales; haustellum vestigial; eye smooth, round, black; front dark brown; labial palpus porrect, dark brown laterally, beige ventrally; patagiun dark brown. ***Thorax and abdomen*** – tegula light gray; mesoscutum dark brown anteriorly, light brown and cream posteriorly; mesoscutellum black and gray; thoracic pleuron beige; legs dark brown on outer surfaces, beige on inner ones; abdominal dorsum dark brown, venter beige.

**Figure 36.**
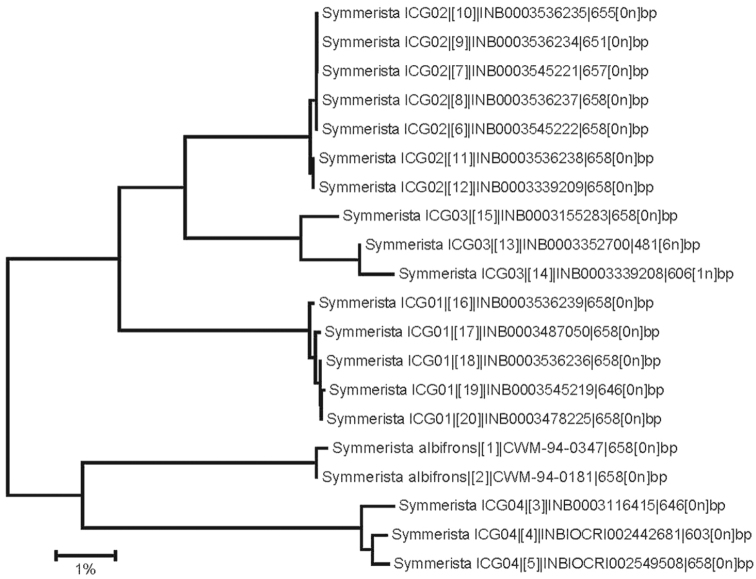
The *Symmerista* species from Costa Rica in a NJ barcoding tree. ICG01, *Symmerista inbioi*; ICG02, *Symmerista luisdiegogomezi*; ICG03, *Symmerista minaei*; ICG04, *Symmerista aura*.

**Wings** – Dorsal FW ground color light gray; mark white cream from the discal cell to apex; costal margin beige until R1; subterminal line black; fringe light gray; dorsal HW light gray, fringe cream (Figs 26, 27) (WL 16.26–17.32 mm).

***Male genitalia*** (Figs 30–34) – T8 wider than long, posterior margin sclerotized and highly irregular; St8 anterior margin with a sclerotized triangular projection in middle, posterior margin sclerotized, irregular, with a rectangular projection at center bearing two highly sclerotic and sharply serrated structures (Fig. 34). Valvae membranous with saccular margin esclerotized, slightly irregular and setose; costal margin smooth, apex rounded, sclerotized and setose; tegumen with margins very sclerotized, narrowed where both arms intersect; uncus slightly concave plate, with papillae and setae in dorsal and ventral edge, with socii elongate, wide at base, narrowed and flattened at apex with papillae and setae in dorsal part (Figs 30, 31, 33); juxta heart shaped; vinculum membranous slightly sclerotized (Fig. 31); proximal part of phallus tube wide and short in base, with a blade-like lateral projection, sharp at distal apex and a small rounded curved projection, distal part of phallus tube robust, sclerotized, with a small bulge on ventral side; proximal part of vesica with a ventral scobinate patch, distal part of vesica bulbous (Fig. 32).

**Female** (Figs 28, 29, 35) Similar to male: ***Head*** – Antenna filiform, antennal shaft light brown with cream scales, scape bearing a long tuft of cream scales; frons and vertex cream; haustellum vestigial; labial palpus mostly cream with black lateral scales. ***Thorax and abdomen*** – tegula light gray; mesoscutum dark brown anteriorly, light brown and cream posteriorly; mesoscutellum black and gray; thoracic pleuron beige; legs dark brown on outer surfaces, beige on inner ones; abdominal dorsum dark brown, venter beige. ***Wings*** – Dorsal FW ground color light gray; mark white cream from the discal cell to apex; costal margin beige until R1; subterminal line black; fringe light gray; dorsal HW light gray, fringe cream (Figs 28, 29) (WL 20.22–21.51 mm). ***Female genitalia*** (Fig. 35) – papillae anales ovoid triangular, setose; posterior apophyses longer and more slender than anterior apophyses; DB sclerotized; CB elongated, membranous and pleated, lacking signum; margin distal of antevaginalis plate very sclerotized and lightly depressed at center, concave to sides, proximal margin convex.

#### Distribution and habitat.

In Costa Rica, *Symmerista aura* has been collected between 1000 to 1400 m on both slopes of the Cordillera de Talamanca (Talamanca Mountain Range) (Fig. 49).

#### Remarks.

DNA barcode paratype male INB0003116415.

MHMXP017-08 | INB0003116415 | *Symmerista aura* | COI-5P:

ACATTATATTTTATTTTTGGGGTTTGAGCTGGGATAGTTGGAACTTCCCTAAGTTTACTAATTCGAGCTGAATTGGGTAACCCTGGATCTTTAATTGGAGATGATCAAATTTATAATACAATTGTAACAGCCCATGCTTTTATTATAATTTTTTTTATAGTTATACCCACCATAATCGGGGGATTTGGTAATTGACTAGTTCCTCTTATATTAGGGGCACCGGATATAGCATTTCCACGTATAAATAACATAAGTTTTTGACTTCTACCCCCTTCTTTAACCCTTTTAATTTCAAGAAGAATTGTCGAAAATGGAGCTGGAACAGGATGAACAGTGTACCCCCCATTGTCATCTAATATTGCTCATGGTGGTAGTTCCGTAGATTTAGCTATTTTTTCACTTCATTTAGTGGAATTTCTTCAATTTTAGGGGCTATTAATTTTATTACAACAATCATTAATATACGTCTTAATAATATATCTTTTGACCAAATACCTTTATTTGTGTGAGCTGTAGGGATTACAGCATTTTTACTTTTACTTTCTTTACCTGTATTAGCTGGAGCTATTACAATATTATTAACTGATCGTAATCTAAACACATCTTTTTTTGATCCCGCTGGAGGAGGAGATCCTATTTTATAC

### Elymiotis tlotzin

Taxon classificationAnimaliaLepidopteraNotodontidae

(Schaus, 1892) comb. n.

Figs 37–45

#### Material examined.

11 males 12 females.

#### Wild-caught adults:

2 Males: INBIOCRI000584965 Costa Rica, Prov. Guanacaste, Bagaces, Ref. Nac. Fauna Silv. R. L. Rodriguez, Estacion Palo Verde, 10.349119-85.352345, 10 m, May 1991, U. Chavarria (INBio). Male: INB0003072431 Costa Rica, Prov. Guanacaste, Bagaces, Pque. Nal. Palo Verde, Sector Palo Verde, 10.366668-85.383266, 0–50 m, 3 May 2000, H. Mendez (INBio). Male: INBIOCRI000386810 Costa Rica, Prov. Guanacaste, Liberia, P. N. Sta. Rosa, Playa Naranjo, 10.80275-85.666572, 0–10 m, May 1991, E. Alcazar (INBio). Male: INBIOCRI002426620 Costa Rica, Prov. Guanacaste, Liberia, Sector Las Pailas, 4.5 Km. SW del Volcan Rincon de la Vieja, 10.776784-85.351913, 800 m, 24 June 1995, K. Taylor (INBio). Male: INB0004065577 Costa Rica, Prov. Guanacaste, Liberia, Santa Rosa Nat. Pk., 10.83641-85.615491, 300 m, 4–6 December 1979, D. H. Janzen (INBio). Male: INB0003319696 Costa Rica, Prov. Guanacaste, Nicoya, P.N. Barra Honda, Sector Barra Honda, 10.169826-85.379137, 50 m, 25–30 December 2000, H. Mendez (INBio). Female: INBIOCRI001184551 Costa Rica, Prov. Guanacaste, Bagaces, P. N. Palo Verde, Estacion Palo Verde, 10.349119-85.352345, 10 m, 20 June 1993, U. Chavarria (INBio). Female: INB0003300310 Costa Rica, Prov. Guanacaste, Hojancha, Z.P. Nosara, Hojancha, R.F. Monte Alto, 10.011248-85.402778, 400 a 500 m, 27 July – 3 August 2000, H. Mendez (INBio). Female: INBIOCRI000674401 Costa Rica, Prov. Guanacaste, Liberia, P.N.Sta. Rosa, Playa Naranjo, 10.802713 – 85.67479, 0–10 m, March 1991, E. Alcazar (INBio). Female: INB0003956448 Costa Rica, Prov. Guanacaste, Nicoya, San Antonio, Humedal Mata Redonda, 10.328094-85.42111, 8 m, 6 July 2005, B. Gamboa, J. Azofeifa, J. Gutierrez, M. Moraga, Y. Cardenas (INBio).

#### Reared from wild-caught caterpillars feeding on foliage of *Zizyphus guatemalensis* (Rhamnaceae):

Male: 94-SRNP-2964 Costa Rica, Area Conservacion Guanacaste, Prov. Guanacaste, Sector Santa Rosa, Estero Naranjo, 10.80426-85.68285, 2 m, 9 June 1994, Gusaneros. Male: 98-SRNP-9137 (COI barcoded), Costa Rica, Area Conservacion Guanacaste, Prov. Guanacaste, Sector Santa Rosa, Area Administrativa, 10.83764-85.61871, 295 m, 14 August 1998, Manuel Pereira. Male: 01-SRNP-17295 (COI barcoded), Costa Rica, Area Conservacion Guanacaste, Prov. Guanacaste, Sector Santa Rosa, Sendero Carbonal, 10.77594-85.65799, 7 m, 2 November 2001, Gusaneros. Male: 06-SRNP-13290 (COI barcoded), Costa Rica, Area Conservacion Guanacaste, Prov. Guanacaste, Sector Santa Rosa, Estero Naranjo, 10.80426-85.68285, 2 m, 31 May 2006, Eilyn Camacho.

Female: 92-SRNP-736 Costa Rica, Area Conservacion Guanacaste, Prov. Guanacaste, Sector Santa Rosa, Vado Nisperal, 10.80212-85.65372, 10 m, 20 May 1992, Gusaneros. Female: 96-SRNP-1331 (COI barcoded), Costa Rica, Area Conservacion Guanacaste, Prov. Guanacaste, Sector Santa Rosa, Sendero Palo Seco, 10.79342-85.6666, 5 m, 31 May 1996, Gusaneros. Female: 96-SRNP-1332 Costa Rica, Area Conservacion Guanacaste, Prov. Guanacaste, Sector Santa Rosa, Sendero Palo Seco, 10.79342-85.6666, 5 m, 2 June 1996, Gusaneros. Female: 98-SRNP-9134 (COI barcoded), Costa Rica, Area Conservacion Guanacaste, Prov. Guanacaste, Sector Santa Rosa, Area Administrativa (adult at light), 10.83764-85.61871, 295 m, 2 August 1998, Guillermo Pereira. Female: 98-SRNP-9137 Costa Rica, Area Conservacion Guanacaste, Prov. Guanacaste, Sector Santa Rosa, Area Administrativa (adult at light), 10.83764-85.61871, 295 m, 14 August 1998, Guillermo Pereira. Female: 01-SRNP-17336 (COI barcoded), Costa Rica, Area Conservacion Guanacaste, Prov. Guanacaste, Sector Santa Rosa, Sendero Carbonal, 10.77594-85.65799, 7 m, 28 October 2001, Gusaneros. Female: 01-SRNP-17336 Costa Rica, Area Conservacion Guanacaste, Prov. Guanacaste, Sector Santa Rosa, Sendero Carbonal, 10.77594-85.65799, 7 m, 28 October 2001, Gusaneros. Female: 07-SRNP-112736 (COI barcoded), Costa Rica, Area Conservacion Guanacaste, Prov. Guanacaste, Sector Santa Rosa, Sendero los Patos (adult at light), 10.82097-85.63323, 251 m, 8 December 2007, H. Cambronero & S. Rios. Female: 11-SRNP-12732 (COI Barcoded), Costa Rica, Area Conservacion Guanacaste, Prov. Guanacaste, Sector Santa Rosa, Area Administrativa (adult at light), 10.83764-85.61871, 295 m, 1 June 2011, Daniel H. Janzen

#### Diagnosis.

**Figures 37–45.**
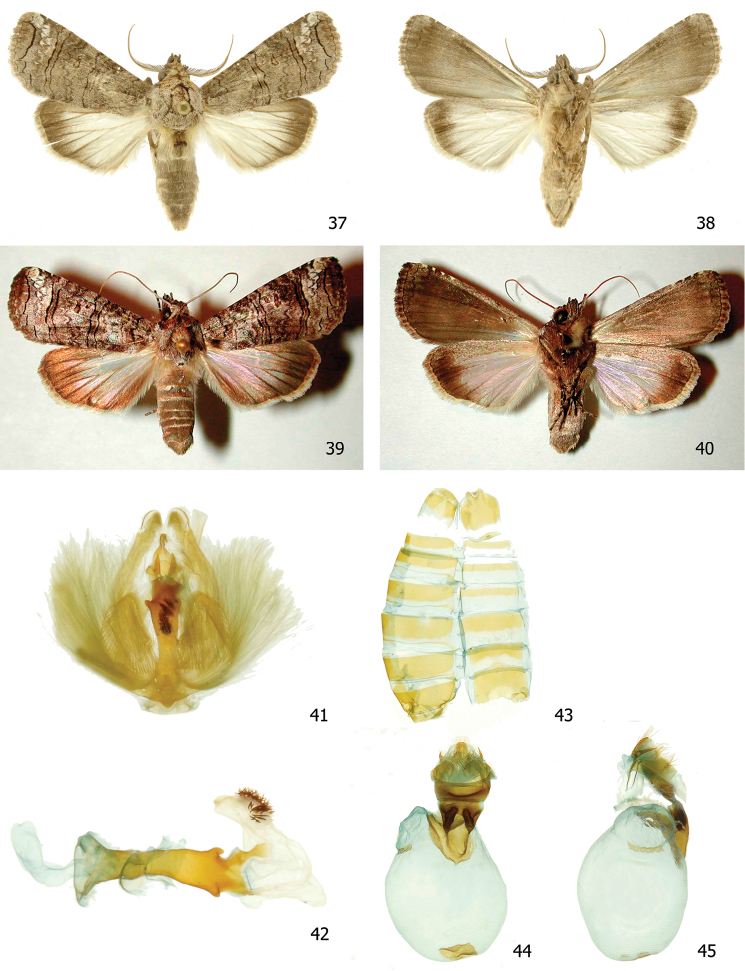
*Elymiotis tlotzin* **37, 38** Male dorsal and ventral INBIOCRI002426620 **39, 40** Female dorsal and ventral 96-SRNP-1332 **41** Male genitalia INBIOCRI002426620 **42** Phallus **43** Male St8 **44, 45** Female genitalia INBIOCRI006744401.

***Adults*** – (Figs 37–40) Medium-sized notodontid moths, FW = 15.42–19.28 mm, females larger than males; male antenna narrowly bipectinate, gradually narrowing to apex, which is simple; antennae of female simple; labial palpus porrect, composed by three segments; haustellum is well developed, ocelli absent; eyes smooth, round. Thorax mostly gray, tegula gray, all scales of the thorax are long and forked; patagium and prothorax with beige and light brown scales. FW costa straight, outer margin almost straight; accessory cell present. ***Male genitalia*** – (Figs 41–43) valvae elongated, sclerotized and mildly setose; sacculus pleats highly developed; uncus thin and long, with apex acute and setose, each socius wide at the base with two thin projections, acute and setose; saccus acute (Figs 41); phallus robust, wide at the base with two single subterminal lateral bumps, vesica long but shorter than phallus, wide at the base, long and thin distally, caltrop cornuti (Fig. 42); T8 rectangular shorter than St8, lateral margins straight, anterior margin simple and membranous, posterior margin slightly concave; St8 wide at anterior margin, posterior margin with a single median depression and two sclerotized small projections (Fig. 43). ***Female genitalia*** (Figs 44, 45) – The papillae anales small, roughly ovoid with elongated dorsal setae; lateral procecess of postvaginal plate with apices acute and sclerotized; posterior apophyses short and thin; lamella postvaginalis rectangular, sclerotized; ostium sclerotized, wide, funnel shape; DB short; CB wide and long, ovoid with surface membranous slightly rough; signum brick shape with a rough surface.

Thiaucourt (2007) “The species was described in the genus *Edema*, cited by DRUCE (1898) in this genus, the species should be now in another genus.” KIRBY (1892) lists *Edema* (Walker, 1855) as a junior synonym of *Symmerista* Hubner, [1821].

The adult lacks the apical white stripe, the male genitalia framework places the genus in the Nystaleini, but it differs from that of *Symmerista* (Plate II, Fig. 10). Male genitalia: uncus with acute and long apex; socci in brackets; valve costa without apical membranous area, pleats highly developed; exopenis with two single subterminal lateral bumps; beam cornuti extensions, obsolete; distal edge St 8 with a single median depression, two sclerotized thickenings near the distal edge of T8.

We propose that *Symmerista tlotzin* should be allocated to the genus *Elymiotis* Walker, 1857 for the following characteristics of the male genitalia: valvae elongated, sclerotized and setose; sacculus pleats highly developed; uncus thin and long, with apex acute and setose; socius wide at the base with two thin projections, acute and setose and caltrop cornuti.

#### Natural history

**Figures 46–48.**
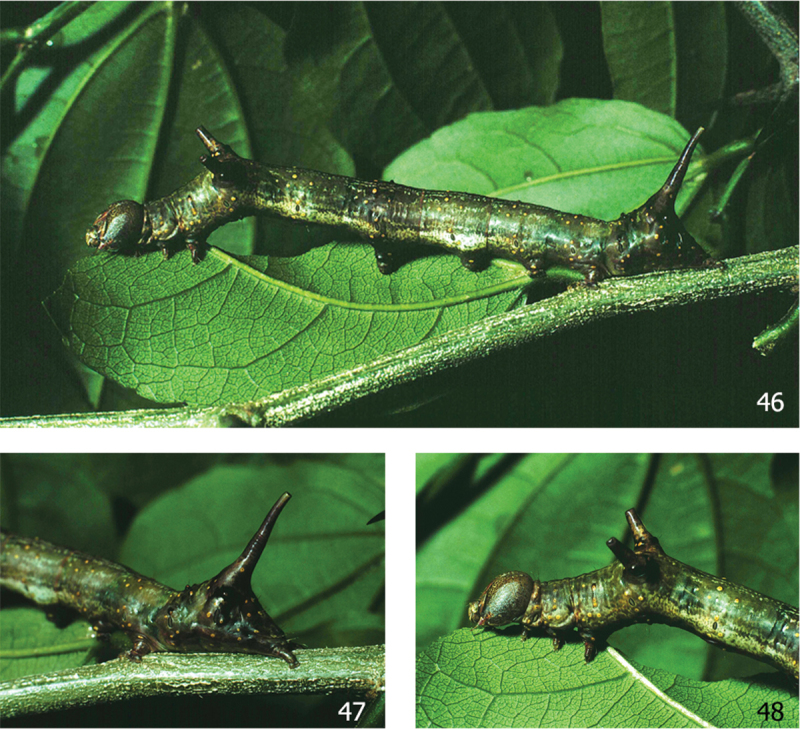
Ultimate instar of *Elymiotis tlotzin* 90-SRNP-1223 on its food plant (Rhamnaceae: *Zizyphus guatemalensis*).

(Figs 46, 47, 48). 33 rearing records from Sector Santa Rosa, ACG.

#### Food plants.

Rhamnaceae, *Zizyphus guatemalensis* Hemsl. (n=33).

#### Parasitoids.

**Eulophidae:** *Euplectrus* (n=1).

#### Distribution and habitat.

**Figure 49.**
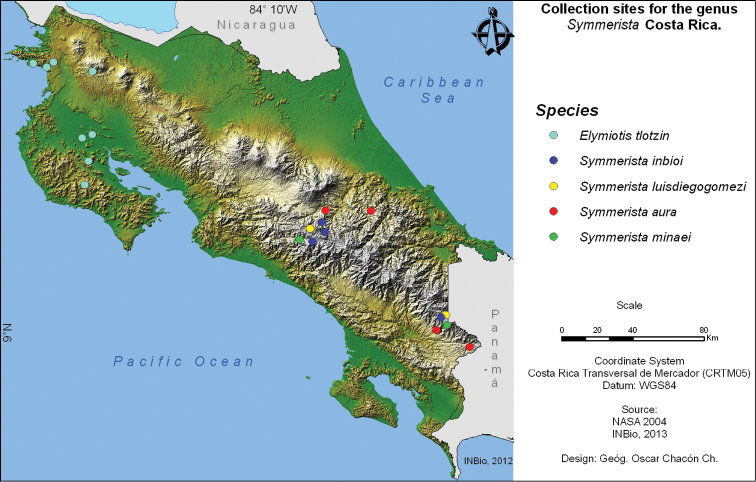
Map of Costa Rican collection sites for the four species of Notodontidae discussed here.

Adult *Elymiotis tlotzin* have been collected in the dry forest ecosystem of Peninsula de Nicoya, and in the dry forests of Sector Santa Rosa and Sector Pailas of ACG, at elevations of 0 to 800 m. (Fig. 49).

#### Remarks.

DNA barcode female 11-SRNP-12732.

MHMYM2073-11 | 11-SRNP-12732 | *Elymiotis tlotzin* | COI-5P:

AACATTATATTTTATTTTTGGAATTTGAGCAGGAATAGTAGGAACTTCTTTAAGTTTATTAATTCGAGCTGAATTAGGAAATCCAGGATCTTTAATTGGTGATGATCAAATTTATAATACTATTGTAACAGCTCATGCTTTTATTATAATTTTTTTTATAGTAATGCCTATTATAATTGGAGGATTTGGAAATTGACTAGTTCCATTAATATTAGGAGCCCCAGATATAGCTTTCCCCCGAATAAATAATATAAGATTTTGACTACTTCCACCCTCACTAACTTTATTGATTTCAAGAAGTATTGTAGAAAATGGAGCAGGAACTGGATGAACAGTTTATCCCCCCCTTTCATCTAA0TATTGCACATAGAGGAAGATCTGTAGATTTAGCAATTTTTTCACTTCATTTAGCTGGTATTTCATCGATTTTAGGAGCTATTAATTTTATTACAACGATTATTAATATACGACTTAATAACATAACTTTTGATCAAATACCTTTATTTGTTTGAGCAGTAGGAATTACAGCTTTTTTATTATTATTATCTTTACCTGTTTTAGCCGGAGCGATTACTATATTATTAACAGACCGTAATTTAAATACTTCATTTTTCGACCCTGCTGGTGGAGGAGATCCAATTCTTTATCAACATTTATTT

## Supplementary Material

XML Treatment for Symmerista

XML Treatment for Symmerista luisdiegogomezi

XML Treatment for Symmerista inbioi

XML Treatment for Symmerista minaei

XML Treatment for Symmerista aura

XML Treatment for Elymiotis tlotzin

## References

[B1] Chacón IA Janzen DH Hallwachs W Sullivan JB Hajibabaei M (2013) Cryptic species within cryptic moths: new species of *Dunama* Schaus (Notodontidae, Nystaleinae) in Costa Rica. In: Schmidt BC Lafontaine JD (Eds) Contributions to the systematics of New World macro-moths IV.ZooKeys 264: 11–45. doi: 10.3897/zookeys.264.4440 23730176 10.3897/zookeys.264.4440 PMC3668374

[B2] Forbes WTM (1948) Lepidoptera of New York and neighboring states. Part 2. Notodontidae. Cornell Agricultural Experiment Station Memoirs 274: 203–237

[B3] Franclemont JG (1946) A Revision of the Species of *Symmerista* Hübner known to occur North of the Mexican border (Lepidoptera, Notodontidae). The Canadian Entomologist 78: 96–103. doi: 10.4039/Ent7896-5

[B4] Gaede M (1934) Notodontidae. Lepidopterorum catalogus. Junk, Gravenhage, 351 pp

[B5] Janzen DH Hallwachs W Blandin P Burns JM Cadiou J Chacon I Dapkey T Deans AR Epstein ME Espinoza B Franclemont JG Haber WA Hajibabaei M Hall JPW Hebert PDN Gauld ID Harvey DJ Hausmann A Kitching I Lafontaine D Landry J Lemaire C Miller JY Miller JS Miller L Miller SE Montero J Munroe E Rab Green S Ratnasingham S Rawlins JE Robbins RK Rodriguez JJ Rougerie R Sharkey MJ Smith MA Solis MA Sullivan JB Thiaucourt P Wahl DB Weller SJ Whitfield JB Willmott KR Wood DM Woodley NE Wilson JJ (2009) Integration of DNA barcoding into an ongoing inventory of complex tropical biodiversity. Molecular Ecology Resources 9 (Suppl. 1): 1–26. doi: 10.1111/j.1755-0998.2009.02628.x 21564960 10.1111/j.1755-0998.2009.02628.x

[B6] Miller JS (1991) Cladistics and classification of the Notodontidae (Lepidoptera: Noctuoidea) based on larval and adult morphology. Bulletin of the American Museum of Natural History 204: 1–230

[B7] Thiaucourt P (2007) *Symmerista* Hübner [1821] Description D’Especes Nouvelles Mesoamericaines (Lepidoptera: Notodontidae). Lambillionea 107: 531–538

[B8] Thiaucourt P Monzón J (2013) *Symmerista brucesuttoni* nouvelle espèce du Guatemala (Lepidoptera, Notodontidae). Bull. Soc. ent. Mulhouse 69(1): 13–14

[B9] Weller SJ (1992) Survey of Adult Morphology in Nystaleinae and Related Neotropical Subfamilies (Noctuoidea: Notodontidae). Journal of Research on the Lepidoptera 31(3-4): 233–277

